# Vitamin D supplementation and serum neurofilament light chain in interferon‐beta‐1b‐treated MS patients

**DOI:** 10.1002/brb3.1772

**Published:** 2020-07-23

**Authors:** Katariina Hänninen, Olli Jääskeläinen, Sanna‐Kaisa Herukka, Merja Soilu‐Hänninen

**Affiliations:** ^1^ Turku University Hospital Neurocenter Turku University Hospital University of Turku Turku Finland; ^2^ Institute of Clinical Medicine‐Neurology University of Eastern Finland Kuopio Finland; ^3^ Neurology Neuro Center Kuopio University Hospital Kuopio Finland

**Keywords:** multiple sclerosis, neuroimaging, NMR, treatment

## Abstract

**Objectives:**

Serum neurofilament light chain (sNfL) is a promising biomarker of MS activity, progression, and treatment response. The aim of the present study was to address whether sNfL concentrations are affected by supplementation of vitamin D and correlate with disease activity in interferon‐beta‐1b (IFNb‐1b)‐treated Finnish MS.

**Materials and Methods:**

Serum samples were available of 32 participants of the Finnish vitamin D randomized controlled trial (17 vitamin D/15 placebo). Serum 25 hydroxyvitamin D was measured using radioimmunoassay and sNfL using single‐molecule array (Simoa). Correlation of sNfL with brain magnetic resonance imaging (MRI) activity, burden of disease (BOD, mm^3^), and disability was assessed at the study baseline and at 52 weeks.

**Results:**

Serum NfL concentrations were similar in the patients randomized to high‐dose vitamin D and placebo at the study baseline and at month 12 follow‐up (*p*‐value). Concentrations of sNfL were higher in patients with Gadolinium‐enhancing lesions in brain MRI: median (95% CI) sNfL was 14.84 (9.9–42.5) pg/ml and 11.39 (8.9–13.2) pg/ml in patients without Gd^+^ lesions (*p* = .0144) and correlated with enhancing lesion volume (Pearson *r* = .36, *p* = .037) at the study baseline but not at week 52. Serum NfL did not correlate with the MRI BOD or disability measured by expanded disability status scale and 25‐foot walk test.

**Conclusion:**

In this small cohort of clinically stable IFN‐treated Finnish MS patients, sNfL levels were similarly low in patients supplemented with high‐dose vitamin D or placebo. Subclinical disease activity in MRI was associated with higher sNfL levels.

## Graphical abstract

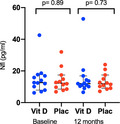

## 1 INTRODUCTION

Neurofilaments are components of the neuronal cytoskeleton released in cerebrospinal fluid (CSF) in association with central nervous system damage. Neurofilament light chain (NfL) is promising as a biomarker in MS activity, progression, and treatment response (Teunissen & Khalil, 2012). The concentrations of NfL in serum (sNfL) and CSF among MS patients are highly correlated (Novakova, Zetterberg, & Sundström, 2017). Vitamin D can also be considered for a serum biomarker of MS. A number of observational studies have shown that low serum levels of 25‐hydroxyvitamin D (25[ OH]D) are associated with clinical relapses or inflammatory disease activity detected in brain magnetic resonance imaging (MRI) in either treatment naïve MS patients or patients treated with immunomodulatory drugs (Ferre, Clarelli, & Aferruzza, 2018; Loken‐Amsrud, Holmoy, & Bakke, 2012; Soilu‐Hänninen, 2005). Among patients with MS treated with interferon‐beta‐1b, low 25(OH)D levels early in the disease course have been shown to be a strong risk factor also for long‐term disease progression (Ascherio et al., 2014). However, conclusive evidence of the benefit of vitamin D supplementation in MS is still lacking, although it is supported by secondary MRI endpoints from several randomized controlled trials (RCTs; Hupperts, Smolders, & Vieth, 2019; Soilu‐Hänninen, Åivo, & Lindström, 2012).

Two previous studies have investigated the effect of vitamin D supplementation on NfL in serum (sNfL) among participants of RCTs with high‐dose vitamin D or placebo supplementation in MS (Holmøy, Røsjø, & Zetterberg, 2019; Smolders, Mimpen, & Oechtering, 2020). Neither of these studies supported and effect of vitamin D on sNfL, but among the Dutch participants of the SOLARIUM study, higher week 48 NfL levels showed a trend for a higher risk of combined unique active lesions in brain MRI (Smolders et al., 2020). Among Swedish MS patients, an inverse association between serum 25(OH)D and CSF‐NfL levels has been shown (Sandberg, Biström, & Salzer, 2016).

We have previously performed an RCT with weekly administration of either 20,000 IU of cholecalciferol or identical placebo capsules in 66 Finnish MS patients (Soilu‐Hänninen et al., 2012). In this paper, we measured serum NfL from serum samples available from 32 patients of the RCT at the study baseline and of *N* = 25 patients (13 vitamin D/12 placebo) at baseline and 52‐week follow‐up. Serum NfL concentrations were measured in duplicate with single‐molecule array (Simoa). Correlation of serum NfL with the number and volume of Gadolinium (Gd)‐enhancing lesions on brain MRI and with MRI burden of disease and disability were assessed. The aim of the present study was to address whether vitamin D supplementation is associated with changes in circulating NfL concentrations in FNB‐1‐b‐treated Finnish MS patients and whether serum NfL concentrations correlate with disability and MRI markers of disease burden and activity.

## 2 METHODS

### 2.1 Study design

The design and clinical and MRI results of the Finnish vitamin D study (cholecalciferol as an add‐on treatment to subcutaneously administered interferon b‐1b [IFNB] for the treatment of MS, NCT01339676) have been published previously (Soilu‐Hänninen et al., 2012). Briefly, 66 RRMS patients aged 18–55 years with IFNb‐1b use for at least 1 months and no neutralizing antibodies to IFNb were detected and baseline 25(OH)D levels below 85 nmol/L, were randomized to either 20,000 IU of cholecalciferol (Swiss‐Caps), or identical placebo capsules, for 52 weeks. All participants gave written informed consent. The study was approved by the ethics committee of Turku University and Turku University Hospital and the National Agency of Medicine, Helsinki, Finland.

### 2.2 Vitamin D and NfL measurements

Serum samples for the 25(OH) D analysis were collected at the study baseline and months 6 and 12 visits, freshly frozen, and stored at −70°C until analyses. Serum 25(OH)D was measured using DiaSorin (Stillwater) ^125^I RIA Kit. The sensitivity of this assay is 4.0 nmol/L, and intra‐assay coefficient of variations (CVs) were <10%. Serum NfL concentrations were measured in duplicate according to the manufacturer's instructions using the NfL advantage kit for Simoa (Catalog number 103186; Quanterix) (Kampman, Steffensen, Mellgren, & Jorgensen, 2012; Rissin, Kan, & Campbell, 2010). Dynamic range of the assay is 0.174–1800 pg/ml, and median intra‐assay CV was 3.5% (interquartile range 1.7%–6.3%, min–max range 0.02%–23%). The Simoa assays were performed in the biomarker laboratory of the Institute of Clinical Medicine‐Neurology, University of Eastern Finland, Kuopio, Finland.

### 2.3 MRI analyses

Brain MRI using a 1.5 Tesla scanner and dual echo T2/PD and postcontrast T1‐weighted sequences covering the whole brain in transverse imaging plane with 3 mm slice thickness was performed at the study baseline and at 52 weeks and centrally analyzed at the Neuroimaging Research Unit, Vita‐Salute University, Milan, Italy, as described previously (Soilu‐Hänninen et al., 2012). Analyses included quantification of the total number of Gd‐enhancing lesions, number of new/enlarging T2/PD lesions and new Gd‐enhancing lesions, T2 lesion volume (BOD) (mm^3^), and T1‐enhancing lesion volume (mm^3^).

### 2.4 Statistical analyses

Statistical analyses were conducted with GraphPad Prism (GraphPad Software) and SPSS (SPSS Inc., version 20.0). Descriptive statistics are provided as mean and standard deviation for normally distributed data and median (95% confidence interval) for skewed variables. Because of non‐normal distribution of the sNfL levels, natural logarithm transformation was used to create normally distributed data. Unpaired *t* test was used to compare differences in the logarithmic sNfL levels between the treatment arms at the study baseline and at week 52 and between patients with or without Gadolinium‐enhancing lesions. Correlation of log‐transformed sNfL levels to other variables was tested using Pearson's correlation. *p*‐values <.05 were considered significant.

## 3 RESULTS

Of the 66 patients in the Finnish Vitamin D study, serum samples were available from 32 patients (17 vitamin D/15 placebo) at the study baseline, *N* = 20 patients (12 vitamin D and eight placebo) at 6 months and of *N* = 25 patients (13 vitamin D/12 placebo) at both the baseline and 52‐week follow‐up visit. MRI data and 25(OH)D results from the study baseline and week 52 visit were available of all these patients. The vitamin D and placebo groups were balanced for the baseline characteristics and did not differ from the total Finnish vitamin D study population. The data of the comparison for the baseline characteristics are not shown. Cohort characteristics of the 32 patients are shown in Table 1. Age, body mass index, disease duration, or smoking status did not significantly correlate with the serum NfL (not shown). Disability during the study was measured by the expanded disability status scale (EDSS) and 25‐foot walk test as described earlier (Soilu‐Hänninen et al., 2012). In the original sample, there was not significant difference in the clinical endpoints at week 52, but there was a trend for less increase in the MRI BOD and significantly less Gd‐enhancing lesions in the vitamin D arm than in the placebo arm at week 52. The distribution of the 52‐week clinical and MRI variables between the original sample, and the subset included in the current study are shown in the Table S1.

**TABLE 1:** Patient characteristics

	Vitamin D group (*N* = 17)	Placebo group (*N* = 15)	*p*‐value
Age (y), mean (*SD*)	38.3 (8.17)	38.5 (7.32)	.91
EDSS^a^ score, median (95% Cl)	2.0 (1.5–3.0)	1.5 (1.0–3.0)	.16
Body mass index, mean (*SD*)	26.2 (5.15)	24.72 (4.96)	.42
Immunomodulatory treatment^a^, *N*	17	15	.60
Smoking, *N*	8	4	.99
25‐hydroxyvitamin D (nmol/L), mean (*SD*)	52.2 (16.86)	58.5 (20.71)	.37
25 FW^b^, mean (*SD*)	4.87 (1.27)	4.90 (1.75)	.67
NfL (pg/mL), median (95% CI)	12.44 (9.9–16.8)	11.86 (8.5–14.8)	.47
T2 BOD, mean (*SD*)	8,452 (10,698)	8,702 (8,743)	
Median (95% Cl)	3,465 (2224–12016)	6,421 (2187–11272)	.71
No. of Gd‐enhancing lesions, mean (*SD*)	0.29 (0.69)	0.20 (0.56)	.89
T1 enhancing lesion volume (mm^2^), mean (*SD*)	30.47 (71.3)	30.20 (89.12)	.93
Disease duration (year), mean			
From onset of symptoms	7.79 (6.94)	7.31 (5.92)	.75
From diagnosis	6.08 (6.22)	4.96 (4.48)	.79
Annual relapse rate^b^, median (95% Cl)	0.49 (0.31–0.66)	0.52 (0.33–0.7)	.67
Interferon therapy (years) Mean, *SD*	2.78	2.61	.67

Note

All patients received interferon β‐1b therapy and had an interferon response measured by MxA test. Annual relapse rate was from 2 years before the study onset.

Abbreviations: BOD, burden of disease, mm^3^; NfL, neurofilament light in serum.

^a^ EDSS, Kurtzke's Expanded Disability Status Scale.

^b^ 25 FW, timed 25‐foot walk test in seconds.

As previously reported (Soilu‐Hänninen et al., 2012), in the group of patients randomized into vitamin D supplementation, the 25(OH)D levels doubled from the baseline to the 52 weeks of follow‐up into a mean of 54.4 nmol/L (*SD* 16.40) at the baseline and 103.7 (*SD* 23.74) nmol/L at week 52, *p* < .0001. In the placebo group, the 25(OH) level at week 52 remained at the low baseline level of 58.5 (*SD* 20.71) nmol/L at the baseline and 57.67 (*SD* 18.78) nmol/L at the week 52. The number patients with vitamin D level >100 nmol/L were 8/13 (61.5%) in the vitamin D group and 0/12 in the placebo group. Median (95% CI) sNfL was similar, 11.66 (6.5–52.8) pg/ml, in the total of eight patients reaching serum 25(OH)D levels above 100 nmol/L at 12 months in comparison with all patients in the whom 25(OH) D was below 100 nmol/L at 12 Months, median (95%) sNflL 11.96 (10.39–16.88) pg/ml, *p*‐value for difference .09. Table 2 shows the sNfL levels by categories of 25(OH)D values when all the 79 sampling occasions at the study baseline, 6 months, and 12 months of the study were included (17 vitamin D, 15 placebo at baseline, 12 vitamin D and 8 placebo at 6 months, and 13 vitamin D, 12 placebo at 52 weeks). At the study baseline, there was a nonsignificant trend for a positive correlation of sNfL and serum 25(OH)D (*r* = .30, *p* = .09), but at month 12 no correlation could be seen (*r* = −.001, *p* = .99).

**TABLE 2:** Serum NfL concentrations by categories of serum 25‐hydroxyvitamin D

25 (OH)D (nmol/L)	NfL (pg/ml)
0‐<25 (*N* = 5)	11.46 (6.18–12.03)
25‐<50 (*N* = 14)	10.31 (7.10–12.91)
50‐<75 (*N* = 27)	13.91 (11.17–17.40)
75‐<100 (*N* = 23)	12.50 (11.03–19.97)
≥ 100 (*N* = 10)	11.08 (8.85–13.62)

Note

Serum samples were collected 79 sampling occasions at the study baseline (32 patients, 17 vitamin D, 15 placebo), 6 months (20 patients, 12 vitamin D, and 8 placebo), and at 12 months (25 patients, 13 vitamin D, 12 placebo). The 25(OH)D values are seasonal. Values are given as median (95% confidence interval). Differences between categories were not significant in unpaired *t* test of log‐transformed sNfL values, and there was no significant correlation between the 25(OH)D values and sNfL in Pearson correlation test.

Abbreviations: 25(OH)D = 25‐hydroxyvitamin D; NfL, neurofilament light.

**FIGURE 1:**
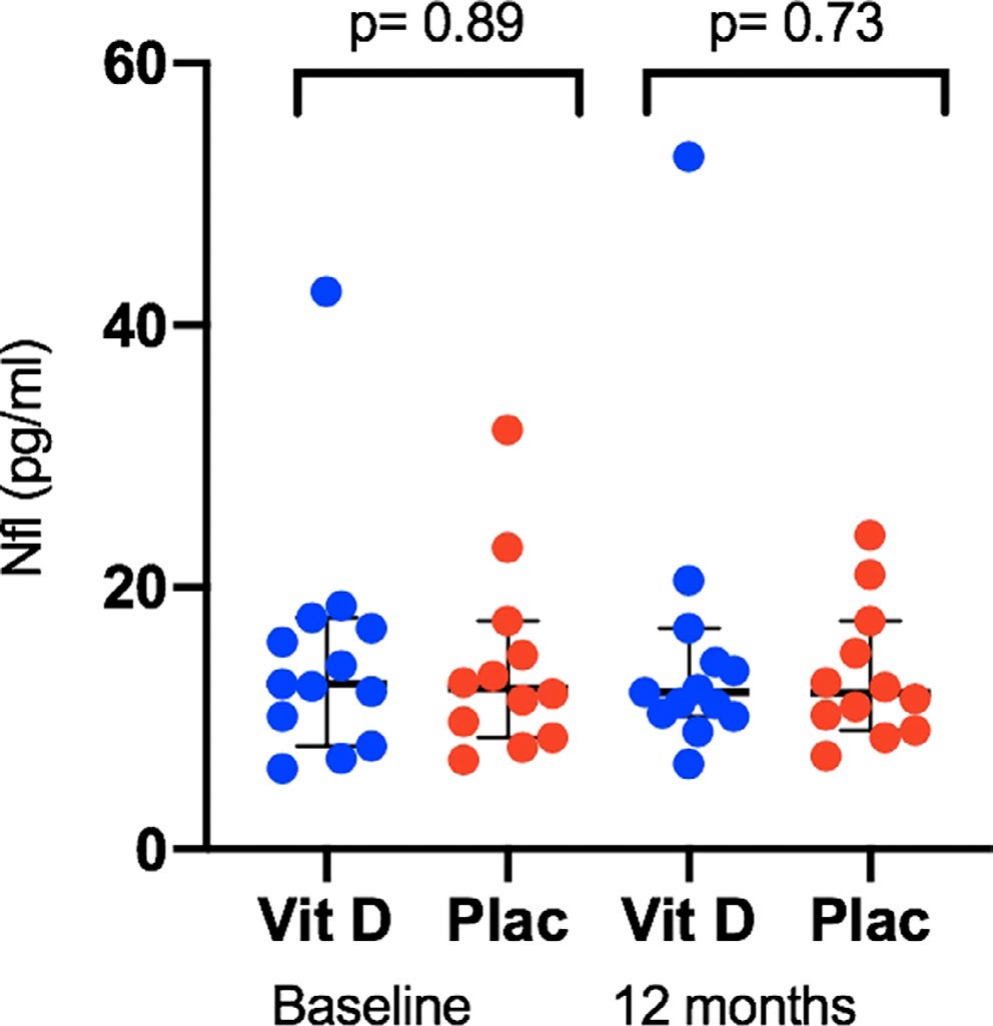
Serum neurofilament light chain (NfL) levels (pg/ml) stratified by treatment and timepoint. Plac, placebo arm; Vit D, vitamin D treatment arm. Bars show median and 95% confidence interval. Significance was tested with unpaired *t* test of log‐transformed sNfL values

Figure 1 shows that there was no significant difference in the serum NfL levels between the vitamin D and placebo groups at the study baseline or at 52 weeks.

None of the 25 patients experienced relapses during the 52 weeks of follow‐up. There was no correlation between the EDSS values and NfL serum concentrations at the study baseline (*r* = .181, *p* = .32) or at week 52 (*r* = −.047, *p* = .86). Levels of sNfL did not correlate with the 25‐foot walk test results at the study baseline (*r* = −.090 *p* = .61) or at 12 months (*r* = −.05, *p* = .8).

**FIGURE 2:**
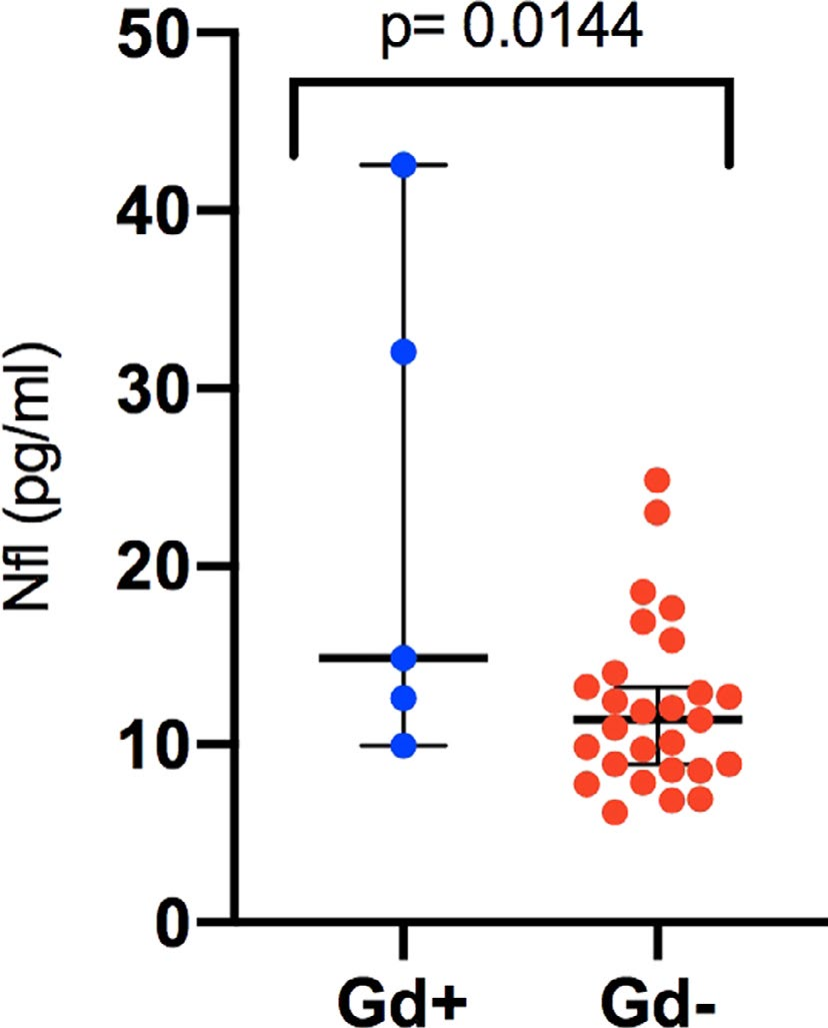
Serum neurofilament light chain (Nfl) levels (pg/ml) in the patients with Gd+ levels and without Gd+ lesions in brain MRI at the study baseline. Bars show median and 95% confidence interval. Significance was tested with unpaired *t* test of log‐transformed sNfL values

A total of five out of 32 patients at the study baseline and four out of 25 patients at 52 weeks had Gd+ lesions in the brain MRI. At the baseline, levels of sNfL were higher in patients with Gd+ lesions than in patients without Gd+ lesions (*p* < .014, Figure 2), but there was no difference at week 52 (not shown). There were only two patients with Gd+‐lesions at week 52 in the vitamin D randomized arm of the whole Finnish Vitamin D study population (*N* = 66 patients), and both of them were included in the current study. The Gd‐enhancing lesion volumes at the study baseline among the five patients with enhancing lesions ranged from 102 to 332 m^3^ and significantly correlated with sNfL (Pearson .36, *p* = .037; Figure S1). At week 52, the enhancing lesions were smaller, ranging from 48 to 52 mm^3^ and did not significantly correlate with the sNfL concentration (not shown). No correlation was seen between MRI BOD and sNfL either at the study baseline (*r* = −.12, *p* = .48) or at month 12 (*r* = .08, *p* = .67).

## 4 DISCUSSION

In this paper, serum NfL concentrations were similar in clinically stable IFN‐beta‐treated Finnish MS patients whether they were supplemented with high‐dose vitamin D or placebo. Patients with subclinical disease activity, emerging as Gadolinium‐enhancing lesions on brain MRI, had higher sNfL levels than patients without MRI activity, and sNfL levels correlated with enhancing lesion volume. Our results concur with the negative results of two previous RCT’s finding no effect of vitamin of supplementation on serum NfL or clinical study endpoints (Holmøy et al., 2019; Hupperts et al., 2019; Kampman et al., 2012; Smolders et al., 2020). In the SOLARIUM study, the patients were also using IFN, but in the Norwegian vitamin D RCT, about half of the patients were not receiving disease‐modifying therapies (Holmøy et al., 2019; Kampman et al., 2012; Smolders et al., 2020). In the latter study, the subgroup of patients not treated with disease‐modifying therapies showed a 30% decrease in sNfL during high‐dose vitamin D supplementation. Thence, it is possible that in untreated MS patients, there is an effect with vitamin D on NfL that is masked by the IFN treatment.

In the study by Holmøy et al from Norway (Holmøy et al., 2019), a surprising positive correlation of sNfL and 25‐hydroxyvitamin D at the study baseline was found in contrast to previously reported association of high serum 25‐hydroxyvitamin D and low CSF‐NfL in Swedish MS patients published by Sandberg et al. (2016). In our study, there was no significant correlation of sNfL and 25‐hydroxyvitamin D either at the study baseline or after vitamin D supplementation, but there were only 10 patients reaching serum levels of 25‐hydroxyvitamin D above 100 nmol/L in our study cohort, and all our patients received IFN therapy. In the Swedish study, only serum 25‐hydroxyvitamin D levels above 100 nmol/L were associated with lower CSF‐NFL levels independently of ongoing MS treatment. It is possible that this explains the diverging results.

All the patients included in our study were using IFNb‐1b and had to have a positive MxA test as a marker of IFN response to be included in the RCT (Soilu‐Hänninen et al., 2012). The treatment with interferon has been shown to lead to a drop in serum NfL in a previous study (Varhaug, Barro, & Bjornevik, 2017). Patients experiencing Gd‐enhancing lesions during IFN therapy had higher serum NfL levels than patients without enhancing lesions in the study by Varhaug et al. (2017). Our results are in line with this, and we also saw a correlation of sNfL concentrations with enhancing lesion volume at the study baseline. We did not have serum samples prior to IFN therapy from our patients nor control patients samples included. The serum NfL levels in our patients without enhancing lesions were low and in the same range as reported by Holmøy et al. (2019) from the Norwegian vitamin D study, and somewhat lower than in the study by Smolders et al. (2020). In a previous study from the biomarker laboratory of the University of Eastern Finland, patients without neurological diseases but with primary psychiatric disorders approximately 15 years older the MS patients in our study had similar NfL concentrations as the MS patients without Gd‐enhancing lesions of our study (Katisko, Cajanus, and Jääskeläinen (2020).

In our cohort of relatively mildly disabled MS patients with short disease durations and no secondary progressive patients included, there was no correlation with sNfL and disability. In a previous Finnish study including older MS patients with longer disease duration and also patients with progressive MS, a correlation of sNfL with disability was found (Högl, Rissanen, & Barro, 2018). These results support sNfL being a marker of also axonal degeneration at least in patients with more advanced MS disease. The MRI T2 lesion volume that we measured from our patients can be considered a cumulative measure of total lesion formation in MS (Chitnis, Gonzales, & Healy, 2018). We did not, however, find any correlation of sNf and the MRI burden of disease in our patients. This may have been driven by the absence of a statistically significant sample size, but may be also explained by the serum samples being collected approximately 7 years after disease onset and 3 years after the initiation of the IFN therapy. In a 10‐year follow‐up of MS patients followed from the disease onset, the strongest correlation between the sNfL and long‐term lesion volume accumulation was observed between NfL samples collected during the first year after disease onset (Chitnis et al., 2018).

The major limitation of our study is the small sample size, since serum samples of only half of the patients included in the parental Finnish vitamin D RCT could be retrieved for the analyses. The strengths are the randomized controlled setting of the study, the MRIs performed with stringent imaging protocol and centrally analyzed in a reference center, and all the patients having an IFN response verified with an MxA test.

## 5 CONCLUSION

In this small cohort of clinically stable Finnish IFN‐treated MS patients, sNfL levels were similarly low in patients supplemented for 52 weeks with either high‐dose vitamin D or placebo. Patients with subclinical disease activity, emerging as Gadolinium‐enhancing lesions on brain MRI, had higher sNfL levels than patients without MRI activity. Our study supports earlier studies suggesting that in clinically stable patients, serum NfL may offer an alternative to MRI monitoring for subclinical disease activity. Although our study did not support vitamin D add‐on therapy affecting serum NfL concentrations, a definite conclusion for or against an effect of vitamin D supplements on serum NfL needs further studies in patients population with high disease activity, or a larger sample size for populations with low disease activity.

## CONFLICT OF INTEREST

M. Soilu‐Hänninen received speaker fees from Biogen, Merck, Novartis, Roche, Sanofi‐Genzyme, and Teva; congress expenses from Biogen, Merck, Roche, Sanofi‐Genzyme, and Teva; and served on advisory boards for Biogen, Merck, Novartis, Roche, Sanofi‐Genzyme, and Teva. K Hänninen, O. Jääskeläinen, and S‐K Herukka declare no conflicts of interest.

## AUTHOR CONTRIBUTION

KH involved in study concept and data collection, statistical analyses, interpretation of data, and drafting of the manuscript; OJ involved in Nf analyses, statistical advice, and critical review of the manuscript, S‐K H involved in NL analyses and critical review of the manuscript; M S‐H involved in study concept and supervision, data collection, drafting, and critical review of the manuscript.

## Supporting information

Fig S1

Table S1

## Data Availability

Data available upon request from the corresponding author upon reasonable request.
