# Supplementary figures and images for: Vitamin D supplementation and serum neurofilament light chain in interferon‐beta‐1b‐treated MS patients
Source: Brain Behav. 2020 Jul 23;10(9):e01772. doi: 10.1002/brb3.1772 (PMC7507359; doi:10.1002/brb3.1772)

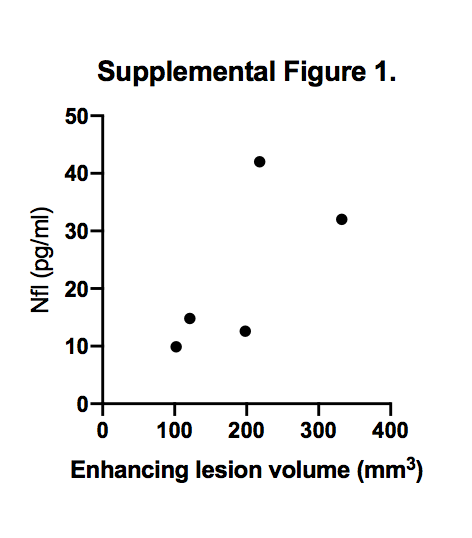

Supplement: Supplementary file 1 — Fig S1 [file BRB3-10-e01772-s001.tiff]
