# Supplementary material for: Vitamin D supplementation and serum neurofilament light chain in interferon‐beta‐1b‐treated MS patients
Source: Brain Behav. 2020 Jul 23;10(9):e01772. doi: 10.1002/brb3.1772 (PMC7507359; doi:10.1002/brb3.1772)
Supplement: Supplementary file 2 — Table S1 [file BRB3-10-e01772-s002.docx]

Supplemental Table 1

12 months MRI/clinical outcomes in the NfL substudy* and in the original Finnish Vitamin D study **

*Vitamin D group (N=17) Placebo group (N=15)

EDSS score, median (range) 2.1 (1.0-3.0) 1.6 (0-3.0)

Number of relapses (mean, SD) 0 0

Number of patients with Gd lesions 2 2

Number of new/enhancing lesions (Mean, SD) 0.5 (0.9) 0.9 (2.2)

MRI BOD median change mm^3^ 48 444

**Vitamin D group (N=34) Placebo (N= 32)

EDSS score, median (range) 2.0 (0-5.5) 1.25 (0-4.0)

Number of relapses (mean, SD) 0.26 (0.51) 0.28 (0.58)

Number of patients with Gd lesions 2 4

Number of new/enhancing lesions Mean (SD) 0.1 (0.2) 0.7 (3.5)

MRI BOD median change mm^3^  83 287

‘ current study

‘’original Finnish Vitamin D study (Soilu-Hänninen et al JNNP 2012)

SD standard deviation

BOD burden of disease
